# MFUM-BrTNBC-1, a Newly Established Patient-Derived Triple-Negative Breast Cancer Cell Line: Molecular Characterisation, Genetic Stability, and Comprehensive Comparison with Commercial Breast Cancer Cell Lines

**DOI:** 10.3390/cells11010117

**Published:** 2021-12-30

**Authors:** Kristijan Skok, Lidija Gradišnik, Helena Čelešnik, Marko Milojević, Uroš Potočnik, Gregor Jezernik, Mario Gorenjak, Monika Sobočan, Iztok Takač, Rajko Kavalar, Uroš Maver

**Affiliations:** 1 Department of Pathology, Hospital Graz II, Location West, Göstinger Straße 22, 8020 Graz, Austria; 2 Faculty of Medicine, University of Maribor, Taborska Ulica 8, 2000 Maribor, Slovenia; lidija.gradisnik@um.si (L.G.); helena.celesnik@um.si (H.Č.); marko.milojevic1@um.si (M.M.); uros.potocnik@um.si (U.P.); gregor.jezernik1@um.si (G.J.); mario.gorenjak@um.si (M.G.); monika.sobocan@gmail.com (M.S.); iztok.takac@um.si (I.T.); rajko.kavalar@gmail.com (R.K.); 3 Faculty of Chemistry & Chemical Engineering, University of Maribor, Smetanova Ulica 17, 2000 Maribor, Slovenia; 4 Division for Gynecology and Perinatology, University Medical Centre Maribor, Ljubljanska Ulica 5, 2000 Maribor, Slovenia; 5 Department of Pathology, University Medical Centre Maribor, Ljubljanska Ulica 5, 2000 Maribor, Slovenia

**Keywords:** breast cancer cell lines, MFUM-BrTNBC-1, MCF-7, MDA-MB-231, MDA-MB-453, hormonal receptors, triple-negative breast cancer

## Abstract

Triple-negative breast cancer (TNBC) is a breast cancer (BC) subtype that accounts for approximately 15–20% of all BC cases. Cancer cell lines (CLs) provide an efficient way to model the disease. We have recently isolated a patient-derived triple-negative BC CL MFUM-BrTNBC-1 and performed a detailed morphological and molecular characterisation and a comprehensive comparison with three commercial BC CLs (MCF-7, MDA-MB-231, MDA-MB-453). Light and fluorescence microscopy were used for morphological studies; immunocytochemical staining for hormone receptor, p53 and Ki67 status; RNA sequencing, qRT-PCR and STR analysis for molecular characterisation; and biomedical image analysis for comparative phenotypical analysis. The patient tissue-derived MFUM-BrTNBC-1 maintained the primary triple-negative receptor status. STR analysis showed a stable and unique STR profile up to the 6th passage. MFUM-BrTNBC-1 expressed EMT transition markers and displayed changes in several cancer-related pathways (MAPK, Wnt and PI3K signalling; nucleotide excision repair; and SWI/SNF chromatin remodelling). Morphologically, MFUM-BrTNBC-1 differed from the commercial TNBC CL MDA-MB-231. The advantages of MFUM-BrTNBC-1 are its isolation from a primary tumour, rather than a metastatic site; good growth characteristics; phenotype identical to primary tissue; complete records of origin; a unique identifier; complete, unique STR profile; quantifiable morphological properties; and genetic stability up to (at least) the 6th passage.

## 1. Introduction

Breast cancer (BC) is the most frequently diagnosed cancer worldwide as of 2020. It is a heterogeneous disease with many subgroups [1,2]. According to the Global Cancer Observatory (GCO) data, an estimated 19.3 million new cancer cases and almost 10.0 million cancer deaths occurred in 2020. From these, female BC (11.7%) has surpassed lung cancer (11.4%) in incidence. BC can be divided into multiple subtypes with different prognoses. These subtypes can be distinguished based on several factors, including histological grade, type and size of the tumour, lymph node metastasis and expression of oestrogen receptor (ER), progesterone receptor (PR) and human epidermal growth factor receptor 2 (HER-2). One of the most common molecular classifications is the division into luminal A-like (ER^+^/PR^+^/HER-2^−^/low Ki67 proliferation index), luminal B-like (HER2^+^) (ER^+^/PR any/HER-2^+^/any value of Ki67), luminal B-like (HER2^−^) (ER^+^/PR^−^ or low/HER-2^−^/high proliferation index Ki67), HER-2-positive (ER^−^/PR^−^/HER-2^+^) and triple-negative (ER^−^/PR^−^/HER-2^−^). Most triple-negative BCs are basal-like breast cancers [3]. The Ki67 proliferation index (PI) is an important parameter in evaluating disease aggressiveness in therapeutic decision making. Nevertheless, up until now, no common validated threshold level has been established. Values at <15% (luminal A)/>15% (luminal B) have been proposed to help discriminate more aggressive subtypes [4]. Triple-negative breast cancer (TNBC) represents 15–20% of all BC cases and has the worst prognosis [2]. Despite a high initial response to chemotherapy (CT), TNBC quickly develops resistance mechanisms. The relative 5-year survival rate for localised TNBC is 91%, for locally advanced it is 65% and for metastatic it is 11% [5]. As therapeutic options are limited, there is an urgent need for new treatment strategies [6]. However, their development requires extensive clinical and preclinical research. Hormone receptor positive and HER-2/Neu amplified cell lines (CLs) and xenografts have been used to accurately predict the response to targeted therapies [7,8,9,10]. One of the most known examples was the finding that anti-oestrogens regulated the growth of tamoxifen-stimulated MCF-7 cells, which subsequently led to trials testing certain drugs (e.g., fulvestrant) and had a major impact on treatment [11,12,13]. Preclinical data from TNBC CLs did not always translate into significant clinical findings [14]. Nevertheless, CLs mirror the original tumours from which they were derived, making them valuable tools for studying molecular aberrations and molecular pathways [14]. With regard to the latter, CLs provide an opportunity to gain further information about the pathophysiology of TNBC and the efficacy of its pharmacotherapeutic treatment [15]. These in vitro model systems are used in various scientific and medical fields, especially in basic cancer research and drug discovery [16,17,18,19,20]. The potential advantages of CLs, particularly in the development of functional disease models, cannot be overlooked [17].

As pointed out by various authors, several naming errors, contaminations, questionable authenticity and reports of questionable inter-laboratory reproducibility and validity have surfaced over the years regarding CL research [18,21,22]. A considerable number of research reports in circulation cited and utilised results obtained by experimenting on contaminated CLs (e.g., HeLa CL; 32.755 articles reporting on research with misidentified cells), as shown by Horbach and Halffman [21]. Ben-David et al. recently described the heterogeneous nature of commonly used cancer CLs [22]. These can quickly change genetically, which plays a part in failed reproducibility and differences in interpreting and/or translating research results.

Bearing this in mind, we believe that there is a need for a new generation of BC CLs—CLs that are appropriately documented and thoroughly characterised according to international standards from the very beginning (see Supplementary document, Table S1) [23,24,25]. This research study aimed to comprehensively describe our patient-derived human TNBC-CL (MFUM-BrTNBC-1) and evaluate it in comparison with commercial BC CLs to obtain a well-defined characterisation (in accordance with international CL cultivation standards), allowing for its efficient use in further TNBC-related studies [26].

## 2. Materials and Methods

### 2.1. Materials for Cell Culturing and Immunocytochemical Staining

Cell lines: For this study, 4 CLs were used: our own primary CL MFUM-BrTNBC-1 [26] and 3 commercially available and widely used BC CLs (MCF-7 [ERα^+^, ERβ^+^, PR^+^, HER2^−^], MDA-MB-231 [Erα^−^, Erβ^−^, PR^−^, HER2^−^], and MDA-MB-453 [Erα^−^, ERβ^+^, PR^−^, HER2^+^]). MFUM-BrTNBC-1 was isolated from a 47-year-old female patient who underwent a standard BC surgical procedure at the University Medical Centre Maribor, Slovenia. Histological examination revealed an invasive carcinoma of no special type (NST), G3, with a triple-negative receptor status and Ki67 PI of 90%. The isolation procedure was described in detail in our previous study [26]. Cell isolation was carried out in a laminar flow cabinet under sterile conditions (Laminar Air Flow Chamber, ISKRA-Pio, Šentjernej, Slovenia). The criteria for selecting the commercial CLs were: CLs with comparatively different phenotypes that can therefore simultaneously serve as internal hormone receptor-positive (MCF-7), HER2 positive (MDA-MB-453) or triple-negative BC controls (MDA-MB-231); well characterised and internationally widely used in research [14,27,28,29,30]; and indexed in international CL databases (Cellosaurus, HyperCLDB, CCLE) [31,32,33,34].

Cell culturing: All the materials and chemicals used were of laboratory grade, suitable for cell culturing. For specific steps of the isolation process and cultivation, labware was additionally autoclaved: Medium Advanced DMEM/F12 (Thermo Fisher Scientific, Waltham, MA, USA), Heat Inactivated Fetal Bovine Serum (Gibco by Thermo Fisher Scientific, Waltham, MA, USA), L-glutamine (Sigma-Aldrich, Merck KGaA, Darmstadt, Germany), penicillin (Sigma-Aldrich, Merck KgaA, Darmstadt, Germany), streptomycin (Sigma-Aldrich, Merck KgaA, Darmstadt, Germany), Phosphate-buffered saline (PBS) (Sigma-Aldrich, Merck KgaA, Darmstadt, Germany), trypsin (Sigma-Aldrich, Merck KgaA, Darmstadt, Germany) and dimethyl sulfoxide DMSO (Sigma-Aldrich, Merck KgaA, Darmstadt, Germany).

Microscopes: Axiovert 40 inverted optical microscope (Zeiss, Oberkochen, Germany) and EVOS FL fluorescence microscope (Thermo Fisher Scientific, Waltham, MA, USA).

Antibodies (Abcam): ER-alfa ((Anti-oestrogen Receptor alpha antibody (EPR4097—ab108398) Alexa Fluor 488), Ex: 495 nm, Em: 519 nm, ab205850 (GFP)), dilution 1:100; PR ((Anti-Progesterone Receptor antibody (YR85—ab32085) Alexa Fluor 647), Ex: 652 nm, Em: 668 nm, ab199455 (Cy5)), dilution: 1:100; HER-2 ((Anti-ErbB2 antibody (EPR19547-12—ab214275) Alexa Fluor 488), Ex: 495 nm, Em: 519 nm, ab225509 (GFP)), dilution 1:200; Ki67 ((Anti-Ki67 antibody (EPR3610—ab92742) Alexa Fluor 488), Ex: 495 nm, Em: 519 nm, ab197234 (GFP)), dilution 1:100; p53 ((Anti-p53 (acetyl K382) antibody (EPR358(2)—ab75754) Alexa Fluor 488), Ex: 495 nm, Em: 519 nm, ab202689 (GFP)), dilution 1:100; ER-beta ((Anti-oestrogen Receptor beta antibody (ERb455—ab212351) Phycoerythrin, Ex: 488 nm, Em: 575 nm, ab205541 (RFP)), dilution 1:50–1:100; Phalloidin ((CytoPainter Phalloidin—iFluor 555 Reagent, Ex: 556 nm, Em: 574 nm, ab176756)).

### 2.2. Methods

**Figure 1:**
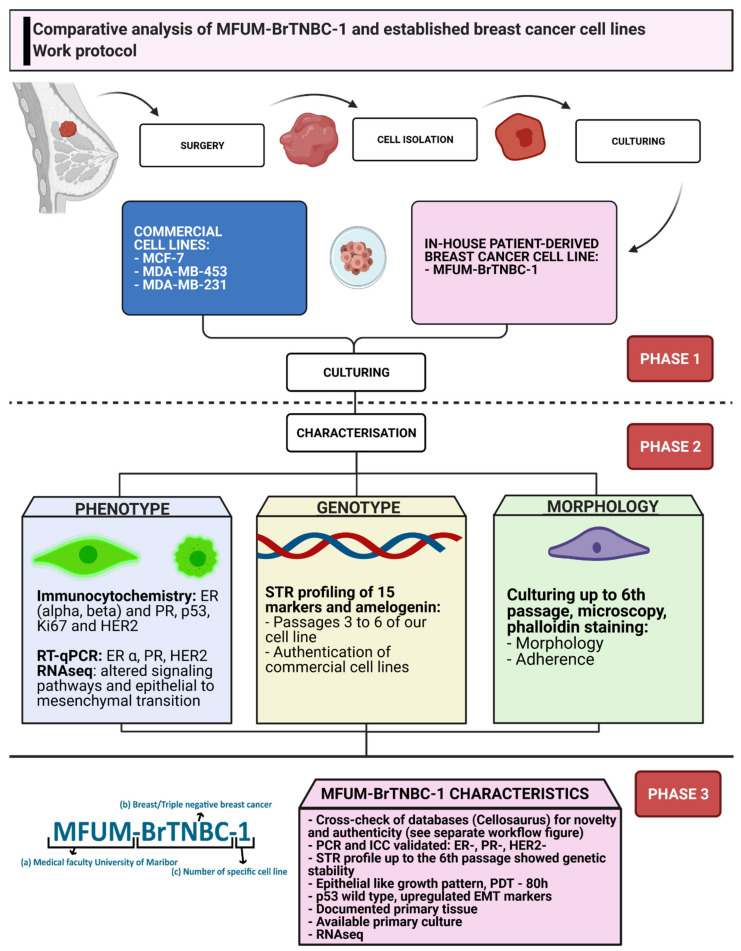
Work protocol of comparative CL analysis. Legend: The picture depicts a stepwise protocol for establishing and characterising MFUM-BrTNBC-1 in adherence to international guidelines and standards. The steps include primary tissue derivation, subsequent isolation, culturing, comparative characterisation and data analysis. ER—oestrogen receptor, PR—progesterone receptor, TNBC—triple-negative breast cancer, STR—short tandem repeat, ICC—immunocytochemistry, RT-qPCR—reverse transcription quantitative real-time PCR.

The steps of our protocol for establishing and characterising MFUM-BrTNBC-1 (schematically shown in Figure 1) employ techniques commonly used to develop functional cell models (models based on primary CLs).

We divided the work protocol into 3 phases (Figure 1). The first phase consisted of surgery, tissue transport, cell isolation and cell culturing of MFUM-BrTNBC-1, and the commercially available CLs. The clinical information about the patient and preparation of all logistic measures were completed beforehand according to the highest ethical standards (the national research ethics committee approval No. “0120-469/2019/6”, the institutional ethics committee approval No. “UKC-MB-KME-3/18” and the signed informed consent from the patient were obtained). After culturing, phase 2 was initiated. Its main purpose was thorough CL characterisation. Each characterisation analysis covered a specific CL feature (e.g., genotype, phenotype, morphology). The analyses consisted of immunocytochemistry (ICC), short tandem repeat (STR) profiling, morphological analysis during culturing via staining and microscopy as well as the evaluation of gene expression by RNA sequencing and qRT-PCR. After completion, all data were gathered and phase 3 commenced. The data were compared, analysed and interpreted. Novelty and authenticity were evaluated via rigorous crosschecking of the available data and international databases.

#### 2.2.1. Phase 1—Pre-Characterisation

##### Cell Isolation

A base cell isolation protocol commonly applied in our laboratories and optimised to address cell type-specific needs has been described in our previous articles [26,35,36]. The multi-step protocol [26] used for the MFUM-BrTNBC-1 isolation can be briefly summarised as follows: (1) after the arrival of the tissue that had been macroscopically selected from the pathologist, the tissue was transferred into two Petri dishes; (2) it was twice rinsed with PBS, which contained additional penicillin and streptomycin; (3) the tissue was then soaked with PBS; (4) necrotic and adipose tissue were macroscopically removed; (5) the tumour tissue was stored in a special Petri dish containing a mixture of 0.25 wt.% trypsin/EDTA; (6) mechanical homogenisation of the tissue was performed with a scalpel (tissue pieces with an average size of 2 mm^3^) while being soaked in trypsin to prevent dehydration; (7) a 1 h incubation period followed; (8) next, the suspension was soaked with Advanced DMEM/F12 s 5 wt.% FBS and split into four 50 mL centrifuge tubes; (9) after centrifugation, the supernatant is discarded and the remaining cell sediment was washed two times with medium (2500 rpm, 15 min); (10) the cells were then resuspended in medium mixed with 5 wt.% FBS, transferred into small Petri dishes for incubation and placed into an incubator.

##### Cell Culturing and Morphological Analysis

For growing and preparing CLs, we relied partially on our own laboratory protocols [35,36] and partially on the protocols supplied by the manufacturer (ATCC). If necessary, the mentioned protocols were adapted to suit the specific needs of the CLs. Details of the culturing conditions are as follows: The cells were cultured in an incubator at 37 °C, 5 wt.% CO_2_. During cell culturing, we kept track of the opacity and colour of the medium. The culturing medium was changed every second day. Growing cells were regularly observed with an Axiovert 40 inverted optical microscope (Zeiss, Oberkochen, Germany) at several magnifications. As briefly (not in-depth) discussed in our previous publication [26], the CL was cultured using 2 different culturing procedures. The difference between the procedures was either allowing for a spontaneous overgrowth of the cancer cells or manual sub-selection of epithelial cells from the beginning, immediately after obtaining the primary cell culture. Both procedures successfully achieved an 80% confluency rate of epithelial cells after one week. The cells exhibited identical morphologies. They were, however, not comparatively phenotypically analysed. Ultimately, the cells gained from the sub-selection method have been utilised for all additional experiments. To determine the population doubling time (PDT), the cells were grown and counted daily for one week. Two time points during the log-phase were used to calculate the PDT value (in hours). Additionally, for further morphological analysis, actin cytoskeleton staining was used. After the last rinse with PBS, the cells were stained with CytoPainter Phalloidin-iFluor 555 Reagent 1:1000 in PBS, containing 1 wt.% BSA and incubated for 90 min in the dark at room temperature. Cells were rinsed three times with PBS and two drops of mounting medium were added. Images were taken using a fluorescence microscope (EVOS FL; ×10 magnification). For the morphological description, we followed the classification into 4 distinct groups (round, stellate, mass, grape-like), according to Kenny et al. [37] Bright-field images obtained by EVOS FL were analysed with the NIH image analysis software ImageJ 1.52 g (NIH, Bethesda, Maryland, USA). Three morphologic parameters (area, perimeter and circularity) were measured and averaged for at least 100 cells per respective CL. Circularity was defined as [4π(cell area)/(cell perimeter)^2^]. These morphologic parameters were used to quantifiably support and ease the classification of the examined CLs, according to Kenny et al. [37] Further details regarding the evaluation protocol using obtained micrographs are shown in the Supplementary document (Figure S1).

#### 2.2.2. Phase 2—Characterisation

##### Microscopy and Immunocytochemistry

We placed round slides with 12 mm in diameter on a P24 plate. Each staining was performed in three wells with a culture population density of 50.000 cells. Immunocytochemical staining was performed for ER (alpha and beta), PR, HER-2, Ki67 and p53 for all 4 CLs. Antibodies were used according to the manufacturer’s guidelines as described in the previous section. After three days of incubation, the medium was removed, the monolayers were rinsed with PBS and the cells were fixed with fixative solution (1:5 in Milli-Q^®^ water) for 15 min at room temperature. They were then rinsed with ice-cold PBS (three times for 5 min). After the last rinse, the cells were permeabilised with the Permeabilization Solution (1:5 in Milli-Q^®^ water) for 10 min at room temperature, followed by 3 more PBS rinses for 5 min. Cells were then incubated with PBS + 1 wt.% BSA + 0.1 wt.% Tween 20 for 30 min at room temperature to block non-specific antibody binding. Diluted primary antibodies were then added in a mixture of PBS + 1 wt.% BSA + 0.1 wt.% Tween 20. The cells were then placed in the refrigerator overnight at 4 °C. The final step of all staining procedures was adding 2 drops of Fluoroshield Mounting Medium with DAPI (4′,6-diamidine-2′-phenylindole dihydrochloride) (Ex: 360 nm, Em: 460 nm, Abcam, ab104139). Images were taken with a fluorescence microscope (EVOS FL; ×10 magnification). The Ki67 PI was also assessed [38]. Briefly, the PI was assessed by the quantification of the proportion of cells positive for the Ki67 antigen. The number of nuclei positive for Ki67 antigen was scored digitally with a bioanalytical image analysis tool (ImageJ) and handpicked individually by eye, then divided by the total number of nuclei. The mean number of total nuclei counted per CL was 100–110, with a minimum of 100 (counted at six distinct regions for each cell type). We evaluated six regions per sample and determined the average with standard error [38,39,40,41,42]. Further details regarding the evaluation protocol using obtained micrographs are shown in the Supplementary document (Figure S2).

##### Extraction of DNA and RNA and Preparation of cDNA

For nucleic acid isolation, cells growing in culture flasks (after reaching confluence as described in Table 1 for respective CLs) were trypsinised, washed twice with PBS and pelleted at 2400 rpm for 15 min at 4 °C. Genomic DNA and total RNA were isolated from cell pellets using the innuPREP DNA/RNA Mini Kit (Analytic Jena, Jena, Germany) according to the manufacturer’s protocol. DNA/RNA purity and integrity were determined by Synergy 2 microplate reader (BioTek, Winooski, VT, USA), Agilent Bioanalyzer 2100 (RNA 6000 Nanochip) (Santa Clara, CA, USA) and agarose gel electrophoresis. RNA (1 microgram, RIN > 9.5) was used for cDNA synthesis with the High-Capacity cDNA Reverse Transcription Kit (Applied Biosystems, Foster City, CA, USA) following the manufacturer’s instructions.

##### STR Profiling

Analysis of 15 STR markers and amelogenin using genomic DNA from MFUM-BrTNBC-1 (third, fourth, fifth and sixth passage) and three commercial human cancer CLs (MCF-7, MDA-MB-231, MDA-MB-453) was carried out at Eurofins Scientific (Eurofins, Ebersberg, Germany).

##### Determination of ER, PR and HER-2 Status by RT-qPCR

Quantitative reverse transcription PCR (RT-qPCR) was performed on the LightCycler^®^ 480 System (Roche, Basel, Switzerland). Taqman gene expression assays (Thermo Fisher Scientific, Waltham, MA, USA) for oestrogen receptor 1 (ER (ESRA) assay Hs01046818_m1), progesterone receptor (PR assay Hs01556702_m1) and erb-b2 receptor tyrosine kinase 2 (HER-2 (ERBB2) assay Hs01001580_m1) were used together with LightCycler^®^ 480 Probes Master (Roche, Basel, Switzerland) according to the manufacturer’s instructions. Relative gene expression levels were calculated by using the 2^−ΔΔCT^ method [43]. For normalisation, the geometric mean of two endogenous controls, Eukaryotic 18S rRNA (VIC reporter) (Applied Biosystems; Foster City, CA, USA) and B2M (Hs99999907_m1, FAM reporter) (Thermo Fisher Scientific, Waltham, MA, USA), was used. CT values over 40 were considered negative.

##### RNA Sequencing

Using RNA extracted in 2.2.2.2., cDNA libraries were prepared with Illumina Stranded mRNA Prep, Ligation kit (Illumina, Inc., San Diego, CA, USA) according to the manufacturer’s instructions. Paired-end read (2 × 74 bp) RNA sequencing (RNAseq) was performed on a NextSeq 550 apparatus (Illumina) with a NextSeq 500/550 Mid Output Kit v2.5 (Illumina, Inc., San Diego, CA, USA). Raw FASTQ data were processed as described previously [44]. RNA expression data of MDA-MB-453, MDA-MB-231 and MFUM-BrTNBC-1 (4th passage) were compared to the MCF-7 epithelial CL.

RNA was further analysed using RNA-seq variant calling pipeline. Raw reads were aligned to reference genome GRCh38 using STAR aligner [45]. PCR duplicates were marked and sorted using PicardTools v2.26.6 (broadinstitute.github.io/picard/; accessed on 13 December 2021. Subsequently, cigar reads spanning splice sites were split, and base quality scores were recalibrated using Genome Analysis Toolkit (GATK) (v4.2.3.0, Broad Institute, Cambridge, MA, USA) [46]. Variant calling was performed using HaplotypeCaller implemented in GATK with a minimum phred scaled confidence threshold of 20.

#### 2.2.3. Phase 3—Post-Characterisation

##### Statistical Analysis, Presentation, and Authenticity/Novelty Check

Statistical analyses were performed using SPSS Statistics 26.00 (IBM Corporation, Armonk, NY, USA). Hormone receptor expression levels were compared between CLs using the Mann–Whitney U-test. HER-2 expression was compared between MFUM-BrTNBC-1 passages by ANOVA with Tukey post hoc tests. Differences were considered statistically significant when *p* < 0.05. Values are presented as the mean ± standard deviation. The figures were prepared using Microsoft Excel (Microsoft Corporation, Redmond, Washington, USA), Adobe illustrator (Adobe Inc., San Jose, CA, USA) and Biorender (Biorender.com; accessed on 14 December 2021). To prove the authenticity and novelty of MFUM-BrTNBC-1, we utilised the Cellosaurus database and STR similarity search tool (CLAST 1.4.4; https://web.expasy.org/cellosaurus-str-search/; accessed on 14 December 2021).

## 3. Results

### 3.1. Phase 1—Pre Characterisation

#### Growth Characteristics and Morphology of MFUM-BrTNBC-1 Cells and Colonies

**Figure 2:**
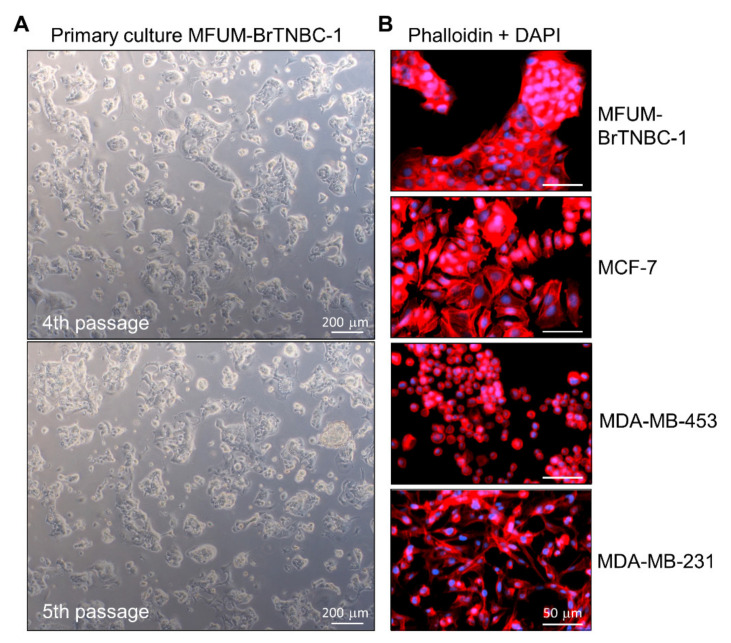
Morphological characteristics of MFUM-BrTNBC-1 CL. Legend: (**A**) The primary MFUM-BrTNBC-1 culture; 4th passage (upper), 5th passage (lower). Images were taken at ×50 magnification on Zeiss Axiovert 40 inverted microscope. Scale bar = 200 μm (**B**) Immunofluorescence images of actin filaments (red) and nuclei (blue) showing cell morphology of MFUM-BrTNBC-1, MCF-7, MDA-MB-453 and MDA-MB-231 (cells were stained by using fluorescent phalloidin conjugate for actin and DAPI for nuclei). Immunofluorescence staining was carried out in triplicate for all CLs, and representative images are shown. Images were taken at ×10 magnification on EVOS FL fluorescence microscope). Scale bar = 50 μm.

During phase 1, we cultured the CLs while investigating their morphological features. MFUM-BrTNBC-1 showed a predominantly round appearance, characterised by the cells coalescing into colonies shaped from round to polygonal. Based on the cell morphology, MFUM-BrTNBC-1 is an epithelial-type cell culture with a patchy appearance (Figure 2A, Table 1). The average time for MFUM-BrTNBC-1 to achieve confluency was from 7 to 10 days. The population doubling time was 80 h. Initially, the cells in suspension that were freely floating displayed a round-like appearance. During the adherence and growth phase, the appearance of the attached cells (which increasingly formed colonies) evolved from roundish to polygonal, characteristic of high-density growth in confluent cell culture (Figure 2A). At low confluence, some cells had cytoplasm-filled projections (pseudopodia) that were no longer visible at higher density and full confluence. When the culture reached a confluent state, the growth continued and the cells began to grow in layers, indicating that they had lost contact inhibition. They were metabolically very active (as evidenced by the change in colour of the medium in just 3 days), which is characteristic of cancer cells [47]. The cells needed to be split after reaching confluence (7–9 days). The morphology of the primary cell culture remained unchanged in consecutive passages (the 4th and the 5th passages 5 days post-seeding are shown in Figure 2A). We cultivated the cells up to the 7th passage, which made it possible to collect enough cells to carry out all planned experiments (e.g., production of cell pellets for STR analysis, see discussion).

**Table 1:** Cell line characteristics.

	MFUM-BrTNBC-1	MCF7	MDA-MB-453	MDA-MB-231
Type *	TNBC [26]	Luminal A [48]	HER-2 [49]	TNBC [50] Basal B
Phenotype *	ER-/PR-/HER-2-	ER+/PR+/HER-2-	ER-/PR-/HER-2+	ER-/PR-/HER-2-
Morphology (group)	Mass	Mass	Grape like	Stellate
Growth (days to reach confluence)	7–9	7	7–9	5–6
PDT	80 h	30–72 h *	38–60 h *	25–48 h *
Oestrogen-a	neg	pos	neg	neg
Oestrogen-b	neg	pos	weakly pos	neg
Progesterone	neg	pos	neg	neg
HER-2	neg	neg	pos	neg
p53 ICC staining	pos	pos	pos	pos
p53 mutational analysis	WT **	WT *	mutated *	mutated *
Ki67 PI (%)	78.30 ± 6.8	87.30 ± 4.3	79.19 ± 5.6	75.98 ± 5.2
Area (µm^2^)	160.94	211.67	90.19	127.01
Perimeter (µm)	51.58	61.92	34.37	66.03
Circularity	0.76	0.70	0.87	0.47

Legend: ER—oestrogen receptor, PR—progesterone receptor, HER-2—human epidermal growth factor receptor, PDT—population doubling time, ICC—immunocytochemistry, WT—wild type, PI—proliferation index. * Based on data from cellosaurus.org [31], synapse.org (Synapse ID: syn2347014). ** Validated by RNA sequencing.

**Figure 3:**
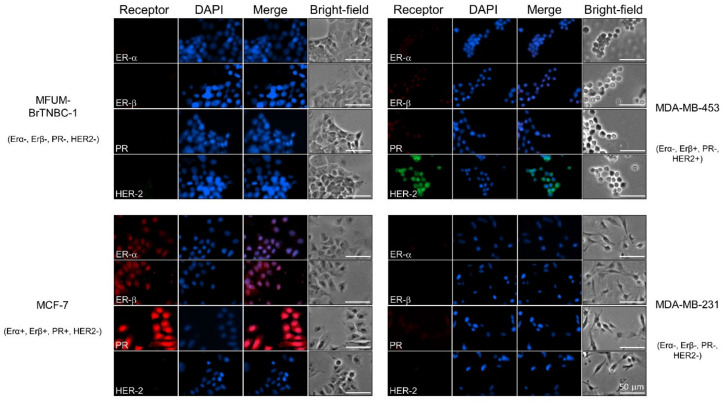
Comparative characterisation of receptor status for human breast cancer cell lines MFUM-BrTNBC-1, MCF-7, MDA-MB-453 and MDA-MB-231. Legend: Immunofluorescence staining was carried out in triplicate for all CLs, and representative images are shown. ER-α—oestrogen α receptor, ER-β—oestrogen β receptor, PR—progesterone receptor, HER-2—human epidermal growth factor receptor 2. Scale bar = 50 mm.

The morphology of individual MFUM-BrTNBC-1 cells was studied in detail at the 4th passage (from this passage on, the CL became visually/morphologically homogenous). This was done by combined immunostaining of actin cytoskeleton (phalloidin staining) and nuclei (DAPI staining) (Figure 2B), followed by light microscopy (Figure 2A and bright-field images in Figure 3). The nuclei were big, middle-centred, round and hyperchromatic. Combined DAPI and phalloidin staining revealed a polygonal shape with actin filaments located at the periphery of the cell. Phenotypic characteristics were calculated using ImageJ, and the data are shown in Table 1 and Supplementary document, Figure S1.

Individual cells showed an average perimeter of 51.58 µm and exhibited an average surface area of 160.94 μm, appeared “round-like” and had a dotted, fine granular and partly pigmented cytoplasm. Values were statistically compared using ANOVA. The statistically highest average cell area values were measured in the MCF-7 CL (211.6 µm^2^), which was morphologically classified as mass-like. MFUM-BrTNBC-1 has the second-highest area value (160.94 µm^2^), followed by MDA-MB-231 (127.01 µm^2^) and finally MDA-MB-453 (90.19 µm^2^). All average cell areas of the examined CLs are significantly different (*p* < 0.05). In the cell perimeter measurements, the highest average cell circumference was observed for MDA-MB-231 (66.0 µm). The statistically highest average value for circularity (0.87) is seen in MDA-MB-453 (grape-like morphological group), followed by 2 CL with a mass-like morphology (MFUM-BrTNBC-1 with a value of 0.76 and MCF7 with 0.70). Finally, MDA-MB-231 (stellate group) has the lowest average circularity value (0.47).

### 3.2. Phase 2—Characterisation

#### 3.2.1. The Immunocytochemical Characterisation of MFUM-BrTNBC-1 Cells and Comparison with Commercial Breast Cancer Cell Lines

MFUM-BrTNBC-1 cells displayed a morphology that was somewhat similar to MCF-7 cells, with cells clustering into closely packed colonies that appeared denser in the case of MFUM-BrTNBC-1 (Figure 2). The cell shapes based on circularity were similar, as confirmed based on quantitative analysis of morphological data (Table 1). MDA-MB-231, despite having a triple-negative phenotype like MFUM-BrTNBC-1, showed morphologically a much more stellate-like growth pattern, with very slim and elongated cells, smaller nuclei and scarce cytoplasm [37], also confirmed from the calculated circularity values (0.47). The cells formed monolayers. In accordance with previous studies, MDA-MB-453 cells had a grape-like growth pattern [37]. MDA-MB-453 cells were round, smaller than MFUM-BrTNBC-1 and had smaller nuclei, as seen from the calculated quantitative morphological data (0.87).

#### 3.2.2. Comparative Analysis of ER-Alpha, ER-Beta, PR and HER-2 Expression in Breast Cancer Cell Lines by Immunofluorescence Staining

Determination of hormone receptor status by measuring the amplification of ER-α, PR and HER-2 is commonly used to classify breast cancer into different subtypes [51]. Using immunofluorescence staining, we detected intracellular expression of two oestrogen receptors (ER-α and ER-β) and PR, and membrane expression of HER-2 in MFUM-BrTNBC-1 and the commercial BC CLs MCF-7, MDA-MB-453 and MDA-MB-231 (Figure 3). MFUM-BrTNBC-1 did not express ER-α, ER-β or PR and lacked HER-2 amplification, which confirmed its triple-negative status. The commercial CLs exhibited staining patterns that were in accordance with previously published studies [27,29,30]. The triple-negative CL MDA-MB-231 showed the same expression pattern as MFUM-BrTNBC-1. Furthermore, MCF-7 (ER^+^ PR^+^ HER-2^−^) had a positive signal for both ER-α and ER-β, a strong signal for PR, but lacked HER-2 amplification. MDA-MB-453 cells displayed strong HER-2 amplification but were negative for ER-α and PR (although they showed a very weak PR signal). As described before, they had a weak positivity for ER-β [52]. The receptor status of all for CLs is depicted in Table 1**,** and staining results can be seen in Figure 3.

#### 3.2.3. Expression of ER-Alpha, PR and HER-2 at the Transcript Level

**Figure 4:**
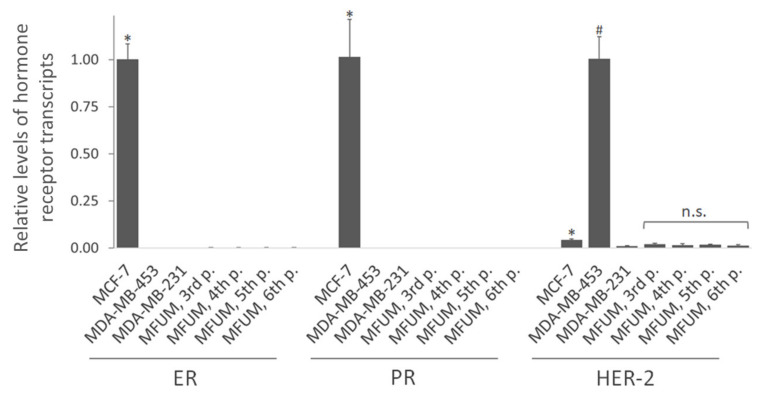
Relative levels of hormone receptor transcripts (ER-α, PR and HER-2) by qRT-PCR. Legend: Relative levels of hormone receptor transcripts in MFUM-BrTNBC-1 and commercial BC CLs MCF-7, MDA-MB-453 and MDA-MB-231 were analysed by qRT-PCR. The level of ER and PR transcripts in MCF-7 cells is presented as 1, while the transcript level of HER-2 is presented as 1 in MDA-MB-453 cells. Comparison of receptor expression in MCF-7 (ER^+^ PR^+^ HER-2^−^) versus other lines: * *p* < 0.05, Mann-Whitney U-test. Comparison of HER-2 expression in MDA-MB-453 (ER^−^ PR^−^ HER-2^+^) versus other lines: # *p* < 0.05, Mann–Whitney U-test. Differences in HER-2 expression between MFUM-BrTNBC-1 passages (MFUM 3rd, 4th, 5th and 6th p.): n.s. (not significant), ANOVA with Tukey post hoc test. Mean ± s.d. is shown.

We also evaluated the receptor status of all CLs at the transcript level by RT-qPCR. (Figure 4). Consistent with the immunofluorescence staining, MFUM-BrTNBC-1 did not express ER-α and PR and lacked HER-2 amplification, confirming its triple-negative status. Its expression pattern was comparable to the commercial triple-negative CL MDA-MB-231. Compared to MCF-7 (ER^+^ PR^+^ HER-2^−^), which also lacks HER-2 amplification, the expression of HER-2 in MFUM-BrTNBC-1 was even lower (*p* < 0.05). It was also substantially lower compared to the CL MDA-MB-453, which has HER-2 amplification (*p* < 0.05). Lack of ER-α/PR expression and HER-2 amplification was confirmed from the third to the sixth MFUM-BrTNBC-1 passage (Figure 4). Differences in HER-2 expression between the passages were not statistically significant. Although immunofluorescence staining showed a surface expression of HER-2 protein only on MDA-MB-453 cells (Figure 3), the more sensitive RT-qPCR method showed high HER-2 expression in MDA-MB-453 cells and a low, insignificant presence of HER-2 mRNA in other CLs [30].

#### 3.2.4. Ki67 and p53 Status

**Figure 5:**
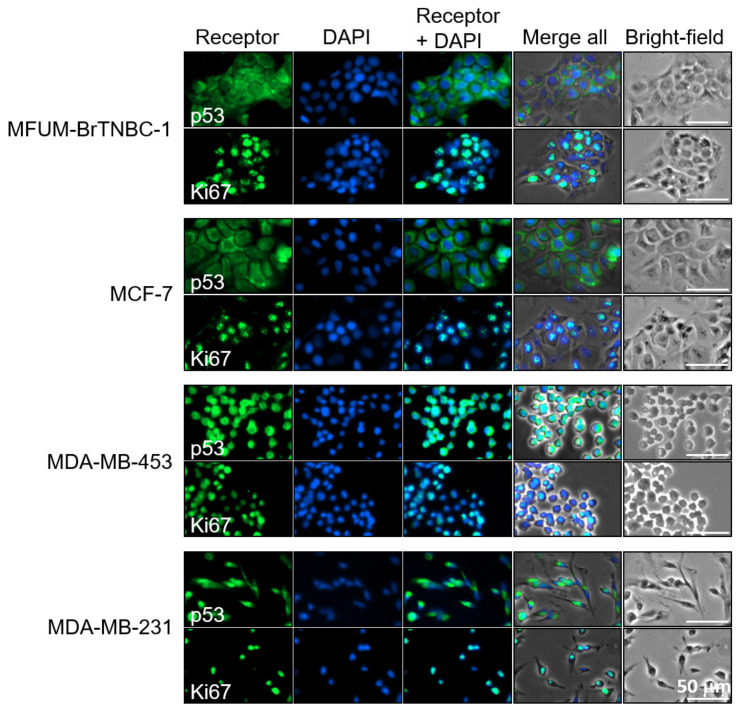
Immunofluorescence staining of p53 and Ki67 in human breast cancer cell lines MFUM-BrTNBC-1, MCF-7, MDA-MB-453 and MDA-MB-231. Legend: Breast cancer cell lines were stained for either p53 (green) or Ki67 (green) and counterstained with DAPI (blue) to visualise the nuclei. The staining was carried out in triplicate for all cell lines and representative images are shown. Scale bar = 50 mm.

We analysed the Ki67 PI of MFUM-BrTNBC-1 and the commercial BC CLs by using immunocytochemistry. In immunofluorescence images (Figure 5), all of the tested CLs displayed positive Ki67 staining, as determined by the green signal in the nuclei. To further analyse the aggressiveness of the tested CLs, the PI was evaluated based on the Ki67 staining. Data shown in Table 1 indicate that the MCF7 CL shows the highest (87.30 ± 4.3%) and MDA-MB-231 (75.98 ± 5.2%) the lowest PI. The calculated Ki67 PI of MFUM-BrTNBC-1 (78.30 ± 6.8%) agrees with that obtained through immunohistochemical staining of the primary source tumour tissue (90%). Exemplary micrographs for the Ki67 calculation can be found in the Supplementary document, Figure S3.

Another important oncological marker is p53, a tumour suppressor protein involved in the cellular response to DNA damage (including induction of cell-cycle arrest, apoptosis and cellular senescence) and tumour metastasis and invasion [53]. Our immunofluorescence images revealed positive p53 staining in all CLs, as determined by the green signal in the nuclei and the cytoplasm. The staining was most robust in MDA-MB-453 cells (Figure 5). MCF-7 has, according to the international databases, a WT p53 status, whereas MDA-MB-231 and MDA-MB-453 are mutated [33]. Based on the staining pattern, MFUM-BrTNBC-1 appeared similar to MCF-7. In addition to immunofluorescence staining, we carried out RNA sequencing of all four CLs and performed variant calling from RNAseq data. This analysis confirmed the known p53 mutations in MDA-MB-231 and MDA-MB-453 and validated the wild type p53-status of MCF-7. Moreover, it revealed that p53 of MFUM-BrTNBC-1 is also of the wild type (Table 2).

#### 3.2.5. Epithelial–Mesenchymal Transition (EMT) of MFUM-BrTNBC-1

**Figure 6:**
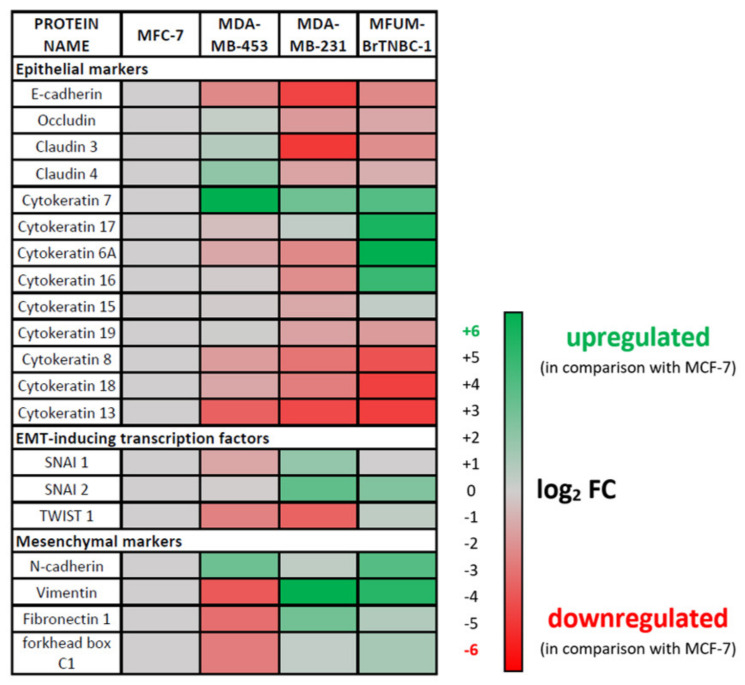
Expression of epithelial–mesenchymal transition (EMT) markers in MFUM-BrTNBC-1, MDA-MB-453 and MDA-MB-231 compared to MCF-7.

The epithelial–mesenchymal transition (EMT) is a cellular programme involved in malignant progression characterised by the remodelling of cell-to-cell and cell-to-extracellular matrix interactions. It is associated with epithelial cells acquiring migratory and invasive properties to become less epithelial and more mesenchymal. The EMT process involves transcriptional changes that promote the mesenchymal fate. In cancer cells, EMT is associated with increased metastatic potential. We have employed RNA sequencing to analyse the expression of EMT markers in MFUM-BrTNBC-1 and the commercial CLs (Figure 6). MFUM-BrTNBC-1 showed upregulation of characteristic mesenchymal markers (such as fibronectin, N-cadherin, vimentin) compared to MCF-7, as well as upregulation of EMT-inducing transcription factors SNAI2 and TWIST1. The expression of EMT-inducing transcription factors is known to inhibit the expression of genes associated with the epithelial state and activate the expression of genes associated with the mesenchymal state. Moreover, the expression pattern of mesenchymal markers in MFUM-BrTNBC-1 resembled that of MDA-MB-231, which has been reported to display representative EMT transition associated with BC metastasis [54].

Compared to MCF-7, MFUM-BrTNBC-1 and MDA-MB-231 cells showed downregulation of characteristic epithelial markers, including E-cadherin, occludin and claudins. Epithelial cells express specific cytokeratins to ensure the resilience of epithelial cell layers to various physical stresses [55]. Several cytokeratins (KRT) that are recognised as epithelial markers, including KRT 8, 18 and 19 [56,57,58], were downregulated in MFUM-BrTNBC-1 and MDA-MB-231 cells. Low expression of KRT19 is known to correlate with poor prognosis in BC patients [57]. On the other hand, KRT7 was upregulated in MFUM-BrTNBC-1 and MDA-MB-231 cells compared to MCF-7. In ovarian cancer cells, KRT7 overexpression was associated with increased proliferation, migration and EMT [59]. KRT 6A, 16 and 17 were upregulated only in MFUM-BrTNBC-1 compared to MCF-7. Upregulated KRT 6A, 16 and 17 have been associated with EMT in cancer cells [60,61,62].

Overall, the epithelial and mesenchymal marker expression suggests EMT changes in MFUM-BrTNBC-1.

#### 3.2.6. Deleterious Mutations and Oncogenic Pathway Signature of MFUM-BrTNBC-1

Cancer development is a complex process in which the accumulation of mutations leads to deregulation of signalling pathways important for cell metabolism, growth and division. Detrimental mutations in MFUM-BrTNBC-1 were identified from RNA sequencing data. A selection of deleterious changes is listed in Table 2.

The mitogen-activated protein kinase (MAPK) pathway plays a role in regulating cellular growth and survival and is frequently dysregulated in human cancers. Increased MAPK pathway activity can be driven by genetic mutations in RAS/RAF family members [63]. Our mutational analysis revealed that MFUM-BrTNBC-1 carries a novel missense mutation (p.G32R) in the BRAF gene, suggesting dysregulation of the MAPK pathway. The analysis also revealed that its KRAS gene is not altered.

Furthermore, we elucidated a few highly deleterious stop-gain mutations in key genes associated with breast cancer in MFUM-BrTNBC-1 (Table 2).

Nucleotide excision repair (NER) is one of the main DNA repair systems. It protects cells against DNA damage, and its deregulation is associated with cancer [64]. MFUM-BrTNBC-1 has a highly deleterious nonsense mutation in the gene XPC (RAD4) involved in NER, indicating a defect in DNA repair.

Epigenetic regulation of gene expression by chromatin remodelling is important for turning genes on or off at specific times. Inappropriate epigenetic modulation can lead to the conversion of normal cells to cancer. MFUM-BrTNBC-1 has a stop-mutation in the ARID1A gene (an SWI/SNF chromatin remodelling gene), which is commonly mutated in cancer (including in MCF-7 and MDA-MB-231 CLs). ARID1A is hypothesised to be tumour suppressive and has been linked to the control of cell cycle/DNA damage checkpoint, regulation of p53 targets and telomerase activation [65].

Dysregulated Wnt signalling is associated with the malignant progression (i.e., proliferation, invasion, metastasis and drug resistance) of BC. In BC, the NRBPI gene, which has a tumour-suppressive role and likely acts through the Wnt/β-catenin signalling pathway, is downregulated [66]. We have identified a stop-mutation in NRBPI in MFUM-BrTNBC-1.

Changes in the phosphoinositide 3-kinase (PI3K) signalling pathway are observed in up to 81% of breast cancer patients [67]. The serine/threonine kinase AKT, a downstream mediator of the PI3K-pathway, regulates cancer progression and metastatic potential. AKT isoforms (AKT1, AKT2, AKT3) play different roles in controlling the migration and invasion of BC cells [67]. We have identified a stop-mutation in AKT3 in MFUM-BrTNBC-1. Downregulation of AKT3 in TNBC cells has been shown to increase migration and metastasis [67]. The PIK3CA gene was not mutated in MFUM-BrTNBC-1, whereas MCF-7 and MDA-MB-453 have missense mutations in this gene.

We also looked into the oxidative stress response in the CLs by examining NFE2L2/NRF2, an important transcription factor that activates cytoprotective genes [68]. None of the CLs had changes in the NFE2L2/NRF2 gene.

Our RNA-seq analysis validated the known mutations in cancer-related genes, GATA3 and CDKN2A, that have been reported for the three commercial CLs.

**Table 2:** Deleterious mutations in MFUM-BrTNBC-1, MCF-7, MDA-MB-453 and MDA-MB-231.

Genes	MFUM-BrTNBC-1	MCF7	MDA-MB-453	MDA-MB-231
p53	Wild-type	Wild-type	Deletion of exons 10 and 11	p.R280K
KRAS	Wild-type	Wild-type	p.T183S ǂ	p.G13D
BRAF	p.G32R ǂ	p.N236Y ǂ	Wild-type	p.G464V
XPC	p.Q829X ǂ	Wild-type	p.R286S ǂ	Wild-type
ARID1A	p.Y2037X ǂ	p.T292P ǂ	Wild-type	p.F1487S ǂ
NRBP1	p.K217X ǂ	p.A222Gfs*3 ǂ	Wild-type	Wild-type
AKT3	p.K294X ǂ	Wild-type	Wild-type	Wild-type
PIK3CA	Wild-type	p.E545L	p.H1047R	Wild-type
NFE2L2/NRF2	Wild-type	Wild-type	Wild-type	Wild-type
GATA3	Wild-type	c.1006dupG	p.L254Q ǂ	Wild-type
CDKN2A	Wild-type	CDKN2A deletion	Wild-type	CDKN2A deletion

Legend: In the commercial CLs, known variants were confirmed, and some new variants (for which the significance is not yet known) were identified. The newly identified variants are marked with ǂ. * stands for termination codon.

#### 3.2.7. STR Profile of MFUM-BrTNBC-1

STR profiling has been an international reference standard for human CLs in terms of authenticity [69,70,71]. To characterise MFUM-BrTNBC-1, its STR profile was determined, including 15 STR markers and Amelogenin (AMEL; for sex determination), as displayed in the Supplementary document, Table S2. To verify genetic stability during in vitro cultivation, STR profiles of the third, fourth, fifth and sixth cell passage of MFUM-BrTNBC-1 were compared. The profiles were identical, indicating STR stability in these passages (Full STR profiles can be found in the Supplementary document, Figure S4).

### 3.3. Phase 3—Post-Characterisation

#### Authenticity and Novelty

Using the CLAST database, we were able to confirm the authenticity of MFUM-Br-TNBC-1 and the commercial CLs. The results using the following settings (Score filter-70%; min markers 13) did not yield any results when crosschecking our own CL out of 7,647 registered human CLs in the database, making it unique. The results for the remaining CLs are as follows: the MDA-MB-231 STR profile had a 100% concordance with published international data [72] and was similar to seven other CLs (similarity score >90%); MCF-7 was also proven genuine [73,74,75] and similar to 30 other CLs; MDA-MB-453 was proven genuine as well [73] and was similar to one other CL. The outline of the approach used to assess the MFUM-BrTNBC-1 authenticity and novelty is shown in the Supplementary document, Figure S5.

## 4. Discussion

This work describes a comparative analysis between a new generation patient-derived TNBC CL (MFUM-BrTNBC-1) isolated in our laboratory and commercially available standard BC CLs, MCF-7, MDA-MB-231 and MDA-MB-453. Based on our results, we claim to have developed an effective isolation, cultivation and characterisation protocol to establish a new generation BC CL.

Compared with other reports, the isolation procedure has proven cost-effective and reproducible over multiple studies and different organic tissue samples [26,35,36]. In the past years, several protocols for isolating cells from breast (cancer) tissue have been developed and reported, together with respective CL characterisation protocols [76,77,78,79,80,81,82]. These isolation protocols differ in complexity as well as repeatability [83,84]. Some of the comparative benefits and drawbacks concerning other protocols are shown in Table 3.

**Table 3:** Comparison of different isolation and characterisation procedures.

Reference	Advantages	Drawbacks
Own (current work, [26])	Simple, cheap, and reproducible isolation protocol Multiple negative and positive control CLs Documentation according to international guidelines Availability of data and tissue STR profiling RNA sequencing (identified oncogenic pathway signature, including gene expression changes and deleterious mutations)	No 3D culturing (Currently) no in vitro drug testing
Weigand et al. [78]	Multiple cell types form the same patient Multiple comparative characterisation procedures Additional 3D culturing STR profiling	More complex and expensive isolation protocol (e.g., two-step tissue digestion procedure etc.)
Widowati et al. [76]	Comparative characterisation with reference CLs One-step enzymatic digestion	Further steps of the isolation procedure were scarcely reported No STR profiling
Finlay-Schultz et al. [82]	BC CLs derived from patient-derived xenografts (PDX) STR profiling	Expensive analysis and preparation methods
Rizwan et al. [77]	Comparative characterisation with reference CLs Additional 3D culturing New ethnic backgroundIn vitro drug sensitivity Growth kinetics	Expensive analysis and preparation methods No STR profiling

During phase one of our experiments, we comparatively analysed MFUM-BrTNBC-1 and the commercial CLs’ morphologies. These characteristics can be seen in Table 1, Figure 2 and Figure 3, as well as from the Supplementary document, Figure S6. The morphology of MFUM-BrTNBC-1 differed from the commercial CLs. A summarised comparison can be seen in Table 1. MDA-MB-231 is a highly aggressive, invasive and poorly differentiated TNBC. The cells are spindle-shaped (long and thin), especially at sub-confluence. Some cells in the culture usually remain rounded. In 3D culture, they can be distinguished by their invasive phenotype, with stellate projections that often bridge multiple cell colonies. They have been classified into the stellate morphological group [37] and display endothelial-like morphology in 3D cultures [85]. MCF7 cells form colonies that may also have round colony outlines, as evidenced through phase-contrast microscopy. This CL has an epithelial-like morphology, and monolayers form dome structures due to fluid accumulation between the culture dish and the cell monolayer [37,48,86]. Lastly, MDA-MB-453 cells form colonies with poor cell–cell contacts and are distinguished by their predominantly grape-like, sometimes elongated, spindly appearance, as well as stellate structures [29,37].

It is known that the architecture of the actin cytoskeleton regulates the motility dynamics of membrane protrusions. It has been demonstrated that mechanical properties of cells (such as cellular stiffness) correlate with several processes, including cell growth, adhesion, differentiation, locomotion, migration, invasion and cancerogenesis [87]. A recent report showed that there might be a correlation between cell cytoskeleton/stiffness and organotropism. The authors found that the cytoskeleton and stiffness of BC cell subpopulations with different metastatic preferences match the mechanics of the metastasised organs [88]. MFUM-BrTNBC-1 exhibited staining of actin filaments at the periphery of the cells (Figure 2). Brinkley et al. investigated different staining patterns in BC CLs and proposed three groups based on the cytoskeletal structure (type I, type II diffuse and type II intermediate) [89]. In MFC-7 cells, actin filaments are distributed mainly around the cell periphery, without other structural elements (such as stress fibres) in the cytoplasmatic region. This was similar in MFUM-BrTNBC-1. The cytokeratin network surrounds the nucleus and extends towards the cell boundary, where F-actin cortical stress fibres are observed. The cellular shape is polygonal, while cells exhibit a more prominent cellular height [90,91]. Furthermore, according to the literature, MCF-7 exhibits a more stable nuclear morphology, and MDA-MB-231 displays a greater malformation profile [90]. In MDA-MB-231, actin stress fibres are evident throughout the cell body, and the nuclear actin cap, a cytoskeletal structure that wraps around the nucleus, can be distinguished (type I) [90]. MDA-MB-453 has been described as having a diffuse actin staining pattern (type II) [89]. Overall, BC CLs display distinct cytoskeletal profiles, with cells of basal B subtype presenting a well-organised stress fibre network throughout the cell body and luminal cells (MCF-7) displaying a primarily peripheral F-actin pattern [90].

We placed MFUM-Br-TNBC-1 into the mass like group based on the quantitative data gained from biomedical image analysis and microscopy. We chose the mass like category, although we were aware that the classification by Kenny et al. describes most TNBC CLs as stellate, which is in line with their invasive nature. However, he also mentioned some outliers (e.g., MCF-10A and MCF-12A), both triple-negative but highly un-invasive CLs that were characterised as a round class by 3D morphology [37]. The gathered biomedical data present another novelty of our article. The comprehensive biomedical image analysis of the microscopic slides helps to evaluate, quantify and correlate the different morphological characteristics (cell area, perimeter and circularity) of the CLs according to the previously established subgrouping systems. Most of the calculated values were comparatively quite different between CLs. Of exception were the measured cell perimeters for MDA-MB -231 and MCF-7 (61.9 µm), which were similar (no statistically significant difference, *p* = 0.31), although the cell area compared between the two was significantly different (MCF-7 are, on average, about 1.6 times larger). As can be seen in the images (Figure 2, Figure 3, Figure 5 and Supplementary material, Figure S1), this can be attributed to the elongated and stretched morphology of MDA-MB-231 cells. The comparison of circularity features seems to reflect the morphological groups (grape-like, mass-like, stellate etc.) even better. Nevertheless, it must be noted that migratory and invasive capacities based on aggregate morphology alone are not always sufficient to predict migratory speed in 2D or invasive capacity in 3D [92].

During phase 2, we comparatively analysed the phenotypical and genotypical characteristics of MFUM-BrTNBC-1. We validated the triple-negative receptor status and thus confirmed the same phenotype as the original tumour while also confirming all phenotypes of the commercial CLs. The Ki-67 PI of our CL (78.29%) was similar to the histological report of the primary tumour (Ki-67 PI—~90%). Furthermore, the Ki-67 PI of the MCF-7 CL was identical to the reported values in the literature (87.30% vs. 90%) [93]. The other CLs had similar Ki-67 PI values. In comparison with reported data, based on the immunohistochemical CL staining, the PI of MDA-MB-231 was lower in our study (75.98% vs. 100%). At the same time, the PI of MDA-MB-453 was similar to published data (79.19% vs. 80%) [39]. Furthermore, the p53 status was investigated. The gene encoding p53 is frequently mutated in human tumours and some also have other mutations that partially hinder the p53 pathway [94]. The interpretation of p53 is somewhat tricky since there are multiple staining patterns of p53 (e.g., IHC staining of endometrial carcinoma, normal/wild-type, complete absence, overexpression and cytoplasmic) [95]. It has been reported that the normal wild-type (WT) pattern can show diverse staining patterns (only some cells to a majority), which is related to the proliferative activity (i.e., higher Ki67–more p53 stained cells) [95]. This might sometimes lead to a misinterpretation as overexpression. The main division regarding p53 status is WT or abnormal [96]. BC CLs with hotspot mutations in the p53-coding gene often show a strong immunofluorescence p53 staining (e.g., ATCC CLs MDA-MB-468, HCC70, SK-BR-3) [97,98]. Based on the staining pattern, MFUM-BrTNBC-1 appeared similar to MCF-7. RNA sequencing analysis confirmed this suspicion and validated the known p53 mutations in MDA-MB-231, MDA-MB-453 and the wild type p53-status of MCF-7.

Furthermore, RNA sequencing of MFUM-BrTNBC-1 revealed an upregulation of characteristic mesenchymal markers similar to MDA-MB-231, which has been reported to display representative EMT transition associated with BC metastasis. During analysis, detrimental mutations in MFUM-BrTNBC-1 were identified. Some of these include a novel missense mutation (p.G32R) in the BRAF gene, suggesting dysregulation of the MAPK pathway, stop-gain mutations in key genes (ARID1A, NRBPI, AKT3), known mutations in cancer-related genes GATA3 and CDKN2A and a nonsense mutation in the gene XPC (RAD4) involved in NER, indicating a defect in DNA repair. In the commercial CLs, known mutational variants were confirmed, and some new variants (for which the significance is not yet known) were identified. The newly identified variants are marked and listed in Table 2.

Phase three consisted of further analysis as well as validating the authenticity and novelty of the CL. There is a variety of TNBC CLs currently reported in the literature. To be exact, a Cellosaurus search for “Triple-negative breast cancer (TNBC) cell line” on 20 September 2021 resulted in 144 entries. However, at least 70% of these CLs were primarily derived from metastatic sites and at least 67% stem from the same patient (e.g., HCC2157, HCC38 etc.) or one parent CL. Furthermore, some of them share very similar STR profiles. A good example is the CL MDA-MB-231 which is based on Cellosaurus, a parent line to at least 43 derived CLs [31] and MCF-7. According to the database, there are 30 other CLs with a higher than 90% similarity STR profile score compared to the MCF-7 CL [31,99]. Based on the presented results, we can claim that MFUM-BrTNBC-1 is authentic and novel (see Supplementary document, Figure S5). Based on the scarce search results, there is a dire need for more genetically diverse CLs of this subtype. This is all the more true as it is known that isolates from metastatic tissue do not always show the same characteristics as the primary tissue despite possible overlaps of some specific phenotypic markers [100]. This can potentially lead to the limited efficiency of chosen pharmacotherapeutic interventions based on such evaluation. It has even been argued that BCs may consist of multiple subtypes within a tumour, potentially due to the plasticity of BC cells. Based on this, the application of single-cell technologies for diagnostic purposes has been proposed when selecting optimal treatment options [101].

MFUM-BrTNBC-1 was isolated from a primary tumour rather than a metastatic site, and the primary cells have been cultivated to complete homogeneity, as previously described [26]. Our workflow offers two specific types of isolation protocols (spontaneous overgrowth or manual sub-cultivation) that have proven to possess identical efficacy in growth confluency dynamics. The morphological analysis via microscopy and phalloidin staining showed that MFUM-Br-TNBC-1 shares morphological similarities with the MCF7 CL [37]. MFUM-Br-TNBC-1 exhibits very good growth characteristics (reaching confluence in ~7 days, PDT–80 h), has the same phenotypical features as the primary tissue, has complete records on the origin of the tissue, is a unique identifier, is a confirmed triple-negative receptor status and has a complete STR profile, all of which will ensure maximal reproducibility wherever and whenever it may be used. Most importantly, it exhibits genetic stability up to (at least) the 6th passage. Furthermore, the CL was proven to be authentic and novel. Genetic stability is crucial to ensure the success and validity of any translational research [71,102]. Moreover, it allows for the cultivation of a high enough number of cells to perform even the most rigorous experiments (e.g., 2 · 10^5 cells for one pellet needed for STR profiling, 10^5^–10^7^ for flow cytometry, 1 × 10^7^ cells per mL for Western blot, 1 × 10^6^ cells for ICH, cryopreservation of cells in multiple ampoules, each containing 1–5 × 10^6^ cells etc.) [71,103]. Our previous report confirmed that the proposed isolation procedure is simple, efficient and cost-effective [26].

## 5. Conclusions

In conclusion, we present an effective protocol for the comparative characterisation of novel CLs against multiple commercially available CLs and offer a new fully characterised authentic TNBC CL for comparative translational studies. We believe that expanding the collection of CLs with new high-quality additions representing the heterogenic nature of BC will steadily shift the medical practice into a patient-centric and individualised medical era.

## Data Availability

The data presented in this study are available in this article. Further information or specific data are available on request from the corresponding authors.

## Supplementary Materials

The following are available online at https://www.mdpi.com/article/10.3390/cells11010117/s1, Figure S1: Quantification of cellular morphologic parameters in different CLs; Figure S2: Ki67 PI evaluation protocol; Figure S3: Micrographs of areas used for Ki67 PI evaluation; Figure S4: Short tandem repeat (STR) genotyping of MFUM-BrTNBC-1; Figure S5: Work protocol for the analysis of authenticity and novelty of our CL; Figure S6: Cultivation process of MFUM-BrTNBC-1. Legend: Legend: (A) Primary suspension with multiple cell types; (B) 12 days post isolation; (C) 1st passage; (D) 1st passage, 17 days after trypsinisation; ^I^ 2nd passage, 4 days after trypsinisation; (F) 3rd passage, 4 days after trypsinisation showing an overgrowth of cancer cells. Images were taken at ×50 magnification on Zeiss Axiovert 40 inverted microscope. Scale bar = 200μm; Table S1: MFUM-BrTNBC-1 datasheet; Table S2: STR profiles of all CLs.
