# Supplementary material for: MFUM-BrTNBC-1, a Newly Established Patient-Derived Triple-Negative Breast Cancer Cell Line: Molecular Characterisation, Genetic Stability, and Comprehensive Comparison with Commercial Breast Cancer Cell Lines
Source: Cells. 2021 Dec 30;11(1):117. doi: 10.3390/cells11010117 (PMC8749978; doi:10.3390/cells11010117)
Supplement: Supplementary file 1 [file cells-11-00117-s001.zip › Table S1.pdf]

Table S1: MFUM-BrTNBC-1 datasheet

CHARACTERISTICS	RESULTS
Cell line name	MFUM-BrTNBC-1
Synonyms	MFUM-BrTNBC-1
Comments	ICC and PCR: ER negative, PR negative, HER2 negative<br>ICC: p53 positive<br>p53 mutational analysis: wild type (WT)<br>Ki67 PI - 78.30 ± 6.8(%)<br>Cell area - 160.94μm^2<br>Cell perimeter - 51.58 μm<br>Cell circularity - 0.76<br>PDT – 80h<br>Upregulation of EMT markers
Deleterious mutations	BRAF p.G32R<br>XPC p.Q829X<br>ARID1A p.Y2037X<br>NRBP1 p.K217X<br>AKT3 p.K294X
Sequence variations	na
HLA typing	na
Genome ancestry	na
Disease	Invasive carcinoma NST, G3<br>TNBC, Ki67 – 90%, androgen receptor negative
Species of origin	Human (homo sapiens)
Sex of cell	Female
Age at sampling	47 years
Category	Cancer cell lines
STR profile	AMEL X, X<br>CSF1PO 11, 11<br>D13S317 13, 14<br>D16S539 11, 12<br>D5S818 12, 12<br>D7S820 9, 11<br>TH01 6, 6<br>TPOX 8, 9<br>vWA 17, 19<br>D8S1179 12, 14<br>D21S11 28, 31.2<br>D3S1358 15, 15<br>D2S1338 22, 24<br>D19S433 13, 14<br>D18S51 12, 16<br>FGA 23, 23
Web pages	https://doi.org/10.1111/tbj.13695
Publications	https://doi.org/10.1111/tbj.13695
Version number	1

Legend: ICC – immunocytochemistry, ER – estrogen receptor, PR – progesterone receptor, WT – wild type.
