# Supplementary material for: MFUM-BrTNBC-1, a Newly Established Patient-Derived Triple-Negative Breast Cancer Cell Line: Molecular Characterisation, Genetic Stability, and Comprehensive Comparison with Commercial Breast Cancer Cell Lines
Source: Cells. 2021 Dec 30;11(1):117. doi: 10.3390/cells11010117 (PMC8749978; doi:10.3390/cells11010117)
Supplement: Supplementary file 1 [file cells-11-00117-s001.zip › Table S2.pdf]

Table S2. STR profiles of all CLs.

STR PROFILE	MFUM-BrTNBC-1	MCF-7	MDA-MB-231	MDA-MB-453
AMEL	X, X	X, X	X, X	X, X
CSF1PO	11, 11	10, 10	12, 13	10, 12
D13S317	13, 14	11, 11	13, 13	12, 12
D16S539	11, 12	11, 12	12, 12	9, 9
D5S818	12, 12	11, 12	12, 12	11, 11
D7S820	9, 11	8, 9	8, 9	10, 10
TH01	6, 6	6, 6	7, 9.3	6, 6
TPOX	8, 9	9, 12	8, 9	10, 10
vWA	17, 19	14, 15	15, 18	17, 18
D8S1179	12, 14	10, 14	13, 13	10, 12
D21S11	28, 31.2	30, 30	30, 33.2	29, 31
D3S1358	15, 15	16, 16	16, 16	15, 15
D2S1338	22, 24	21, 23	20, 21	23, 24
D19S433	13, 14	13, 14	11, 14	13, 14
D18S51	12, 16	14, 14	11, 16	15, 20
FGA	23, 23	23, 25	22, 23	18, 23

†STR profiling was used to authenticate the ATCC® lines used in this study
