# Supplementary figures and images for: MFUM-BrTNBC-1, a Newly Established Patient-Derived Triple-Negative Breast Cancer Cell Line: Molecular Characterisation, Genetic Stability, and Comprehensive Comparison with Commercial Breast Cancer Cell Lines
Source: Cells. 2021 Dec 30;11(1):117. doi: 10.3390/cells11010117 (PMC8749978; doi:10.3390/cells11010117)

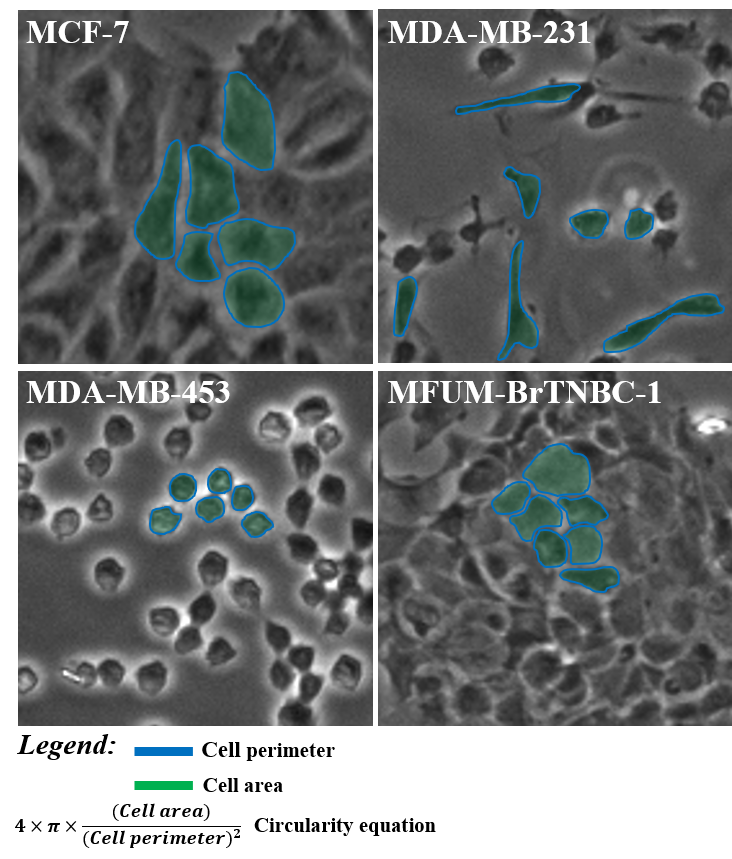

Supplement: Supplementary file 1 [file cells-11-00117-s001.zip › Figure S1.tif]

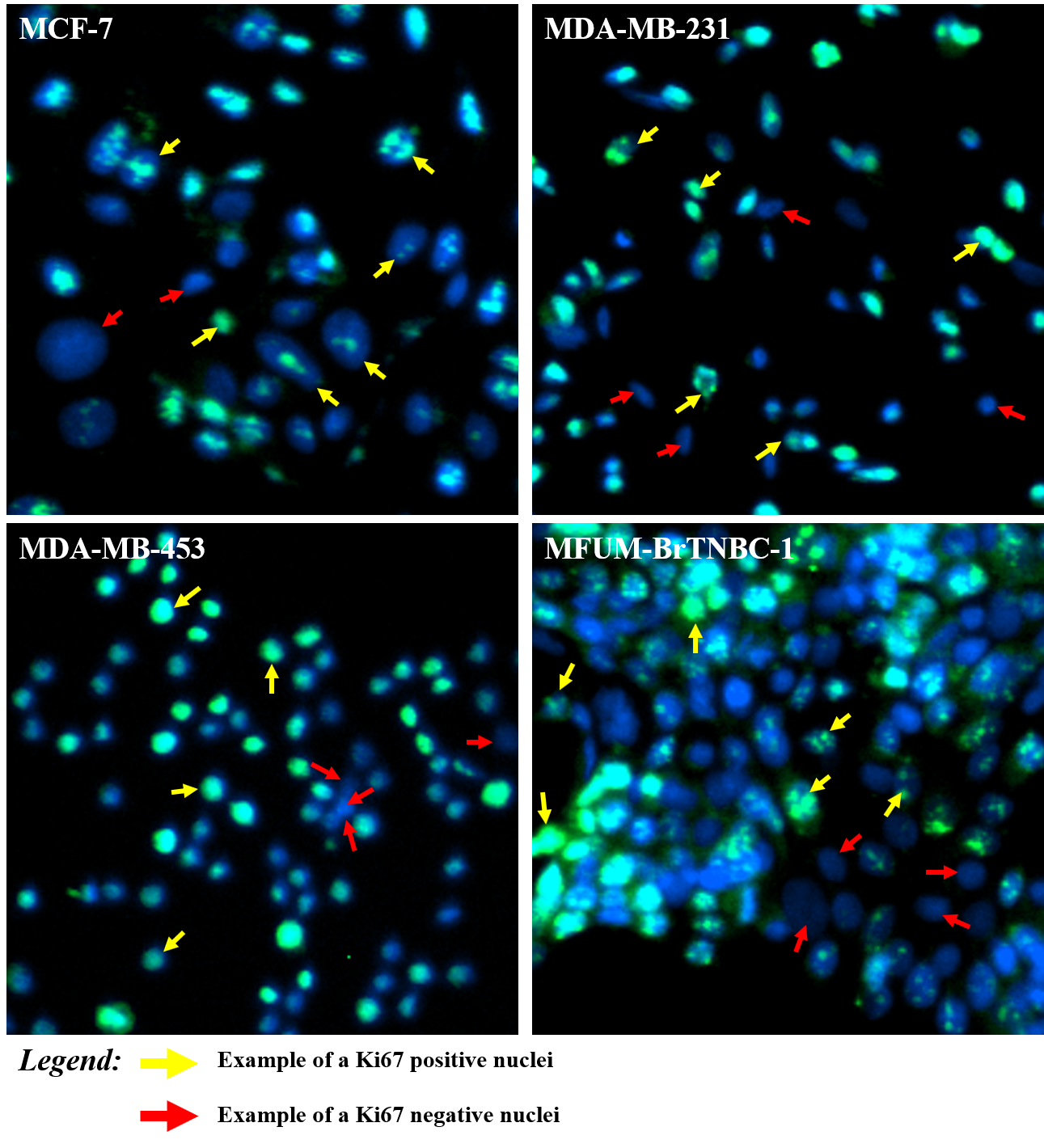

Supplement: Supplementary file 1 [file cells-11-00117-s001.zip › Figure S2.tif]

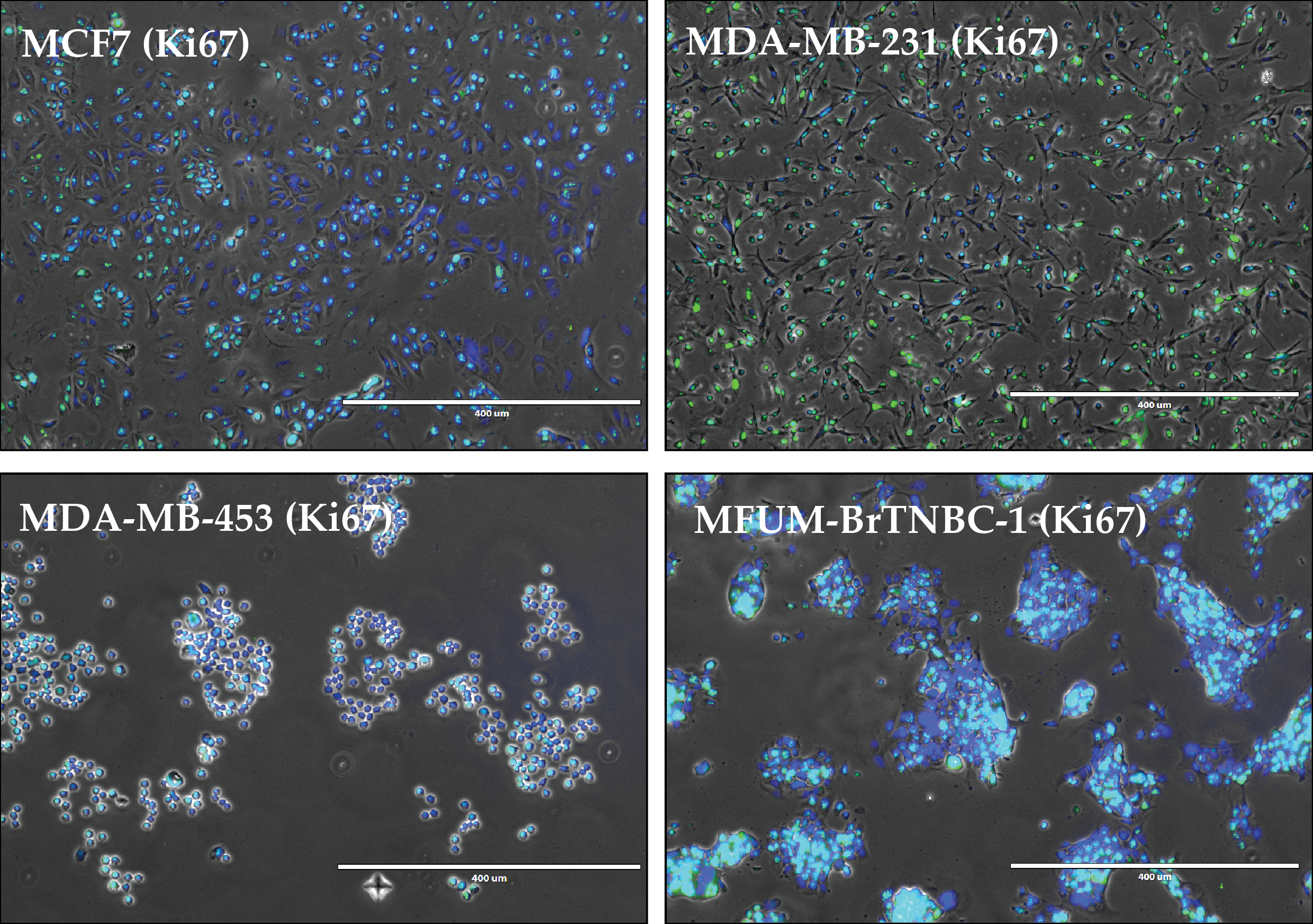

Supplement: Supplementary file 1 [file cells-11-00117-s001.zip › Figure S3.png]

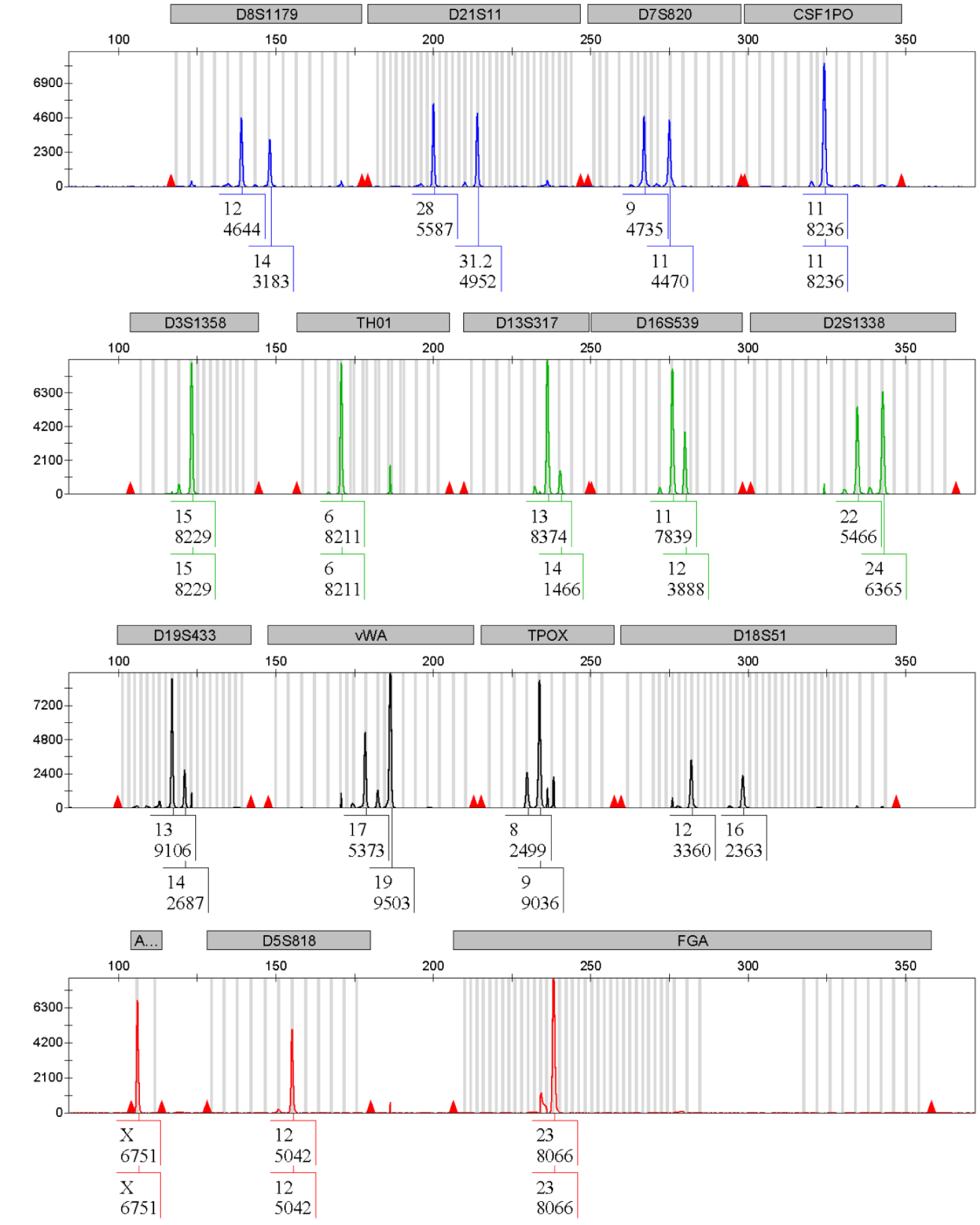

Supplement: Supplementary file 1 [file cells-11-00117-s001.zip › Figure S4.png]

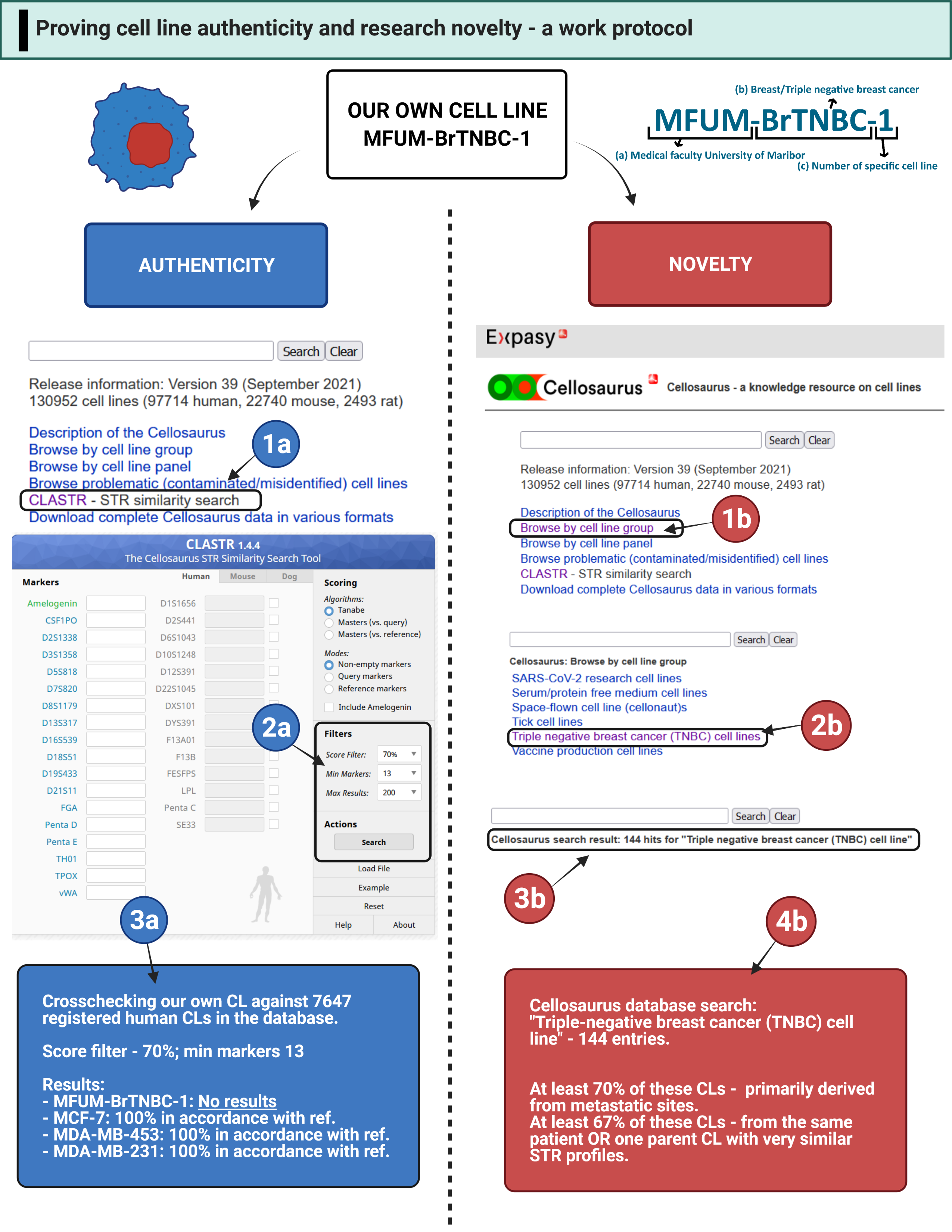

Supplement: Supplementary file 1 [file cells-11-00117-s001.zip › Figure S5.png]

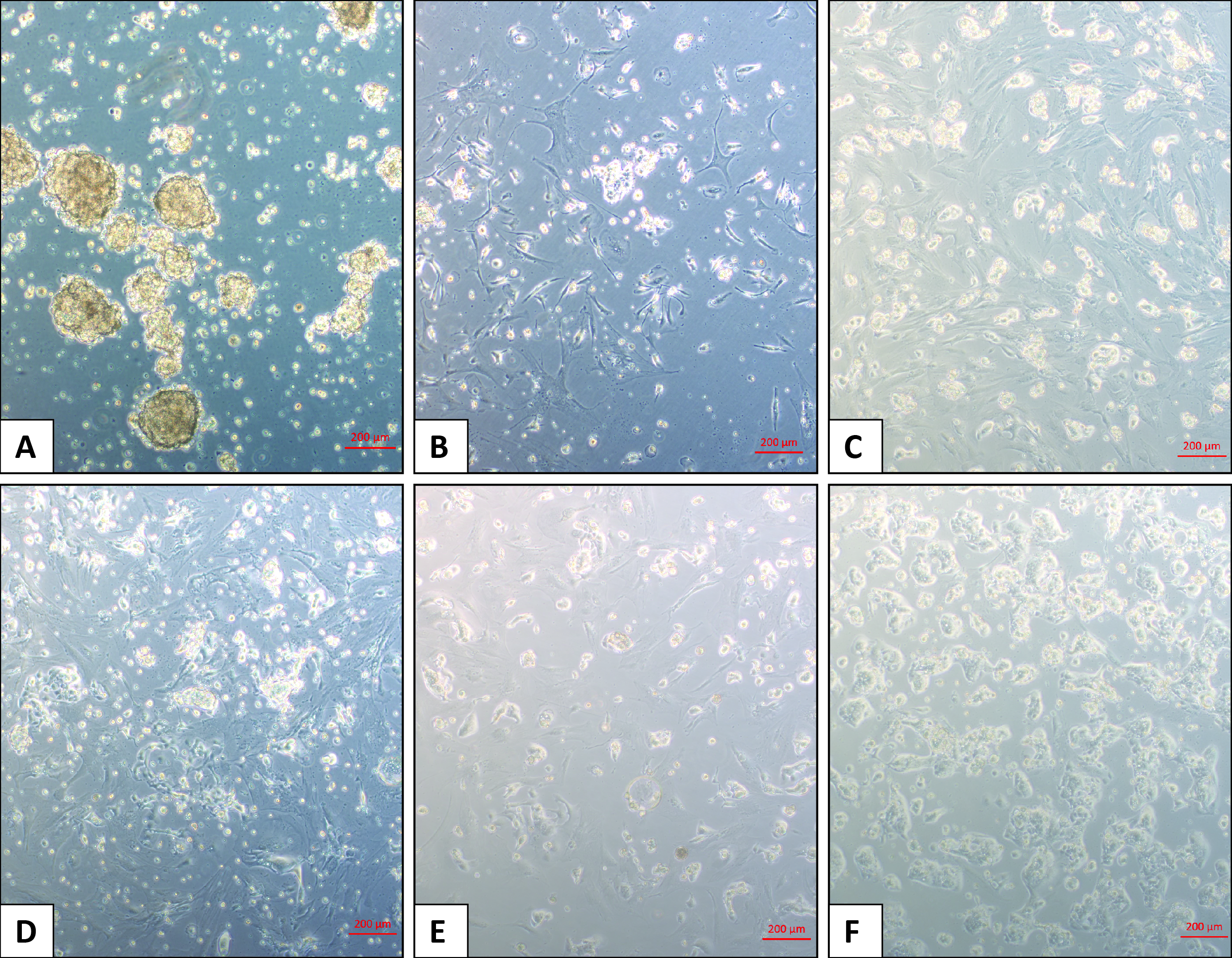

Supplement: Supplementary file 1 [file cells-11-00117-s001.zip › Figure S6.jpg]
